# Social Participation and the Prevention of Functional Disability in Older Japanese: The JAGES Cohort Study

**DOI:** 10.1371/journal.pone.0099638

**Published:** 2014-06-12

**Authors:** Satoru Kanamori, Yuko Kai, Jun Aida, Katsunori Kondo, Ichiro Kawachi, Hiroshi Hirai, Kokoro Shirai, Yoshiki Ishikawa, Kayo Suzuki, The JAGES Group

**Affiliations:** 1 Tokyo Medical University, Department of Preventive Medicine and Public Health, Tokyo, Japan; 2 Human Resource Management Department, ITOCHU Techno-Solutions Corporation, Tokyo, Japan; 3 Physical Fitness Research Institute, Meiji Yasuda Life Foundation of Health and Welfare, Tokyo, Japan; 4 Department of International and Community Oral Health, Tohoku University Graduate School of Dentistry, Miyagi, Japan; 5 Center for Preventive Medical Science, Chiba University, Chiba, Japan; 6 Center for Well-being and Society, Nihon Fukushi University, Aichi, Japan; 7 Department of Social and Behavioral Sciences, Harvard School of Public Health, Boston, Massachusetts, United States of America; 8 Faculty of Engineering, Department of Civil and Environmental Engineering, Iwate University, Iwate, Japan; 9 Department of Human Sciences, School of Law and Letters, University of the Ryukyus, Okinawa, Japan; 10 Department of Health and Social Behavior, School of Public Health, The University of Tokyo, Tokyo, Japan; 11 Department of Social Policies, Aichi Gakuin University, Aichi, Japan; Federal University of Rio de Janeiro, Brazil

## Abstract

**Background:**

We examined the relationship between incident functional disability and social participation from the perspective of number of types of organizations participated in and type of social participation in a prospective cohort study.

**Method:**

The study was based on the Aichi Gerontological Evaluation Study (AGES) Cohort Study data. We followed 13,310 individuals aged 65 years or older for 4 years. Analysis was carried out on 12,951 subjects, excluding 359 people whose information on age or sex was missing. Social participation was categorized into 8 types.

**Results:**

Compared to those that did not participate in any organizations, the hazard ratio (HR) was 0.83 (95% CI: 0.73–0.95) for participation in one, 0.72 (0.61–0.85) for participation in two, and 0.57 (0.46–0.70) for participation in three or more different types of organizations. In multivariable adjusted models, participation in the following types of organization was protective for incident disability: local community organizations (HR = 0.85, 95% CI: 0.76–0.96), hobby organizations (HR = 0.75, 95% CI: 0.64–0.87), and sports organizations (HR = 0.64, 95% CI: 0.54–0.81).

**Conclusion:**

Social participation may decrease the risk of incident functional disability in older people in Japan. This effect may be strengthened by participation in a variety of different types of organizations. Participating in a local community, hobby, or sports group or organization may be especially effective for decreasing the risk of disability.

## Background

Determining factors for preventing incident functional disability is a critical goal for societies confroting in rapid population aging, including Japan. Social relationships have been suggested as one factor that helps lower the risk of functional disability, [1]. Conversely, poor social relationships have been shown by meta-analysis to raise mortality risk, [2]. Social relationships encompass social network ties, the exchange of social support, as well as social participation, i.e. participation in civic groups that an individual can join, regardless of occupation or family situation. Promoting social participation is one of the key proposals of ‘Active Aging’ (the World Health Organization's policy framework), [3].

Previous studies have suggested that social participation may lower the risk of all-cause mortality, [4]–[8], cardiovascular mortality, [7], all circulatory system disease mortality, [8], non-cancer and non-circulatory system disease mortality, [8], acute myocardial infarction, [9], incident disability, [10], [11], motor decline, [12], cognitive decline, [7], [13], and depressive symptoms, [14], [15]. Social participation may thus be health promoting across a wide range of outcomes. When creating policies for promoting social participation, it is necessary to determine what forms of participation are effective. However, most of the above studies only examined broad social participation in any sort of organization or they lumped participation in various types of organizations together, and very few were able to look at the relationships with intensity of involvement or types of organizations.

Studies that did examine the effects of participation according to different types of organizations showed that participation in multiple organizations may have a protective effect on depression, [15], well-being, [16] and oral health status, [17]. In addition, people who maintain a role in social organizations (e.g. secretary, treasurer, etc) experience a lower risk of depression,[18]. It is possible that participation in a diversity of organizations increases the number of roles, thereby reducing the risk of incident functional disability, but this theory has yet to be examined.

One study was conducted on participation in a sports organization to examine the effect on risk of incident functional disability, [19]. Those that participated in a sports organization were less likely to require long-term care than those who exercised individually (i.e. on their own), even if they did so once a week or more. Moreover, as long as they participated in a sports organization, the frequency of exercise appeared to make little difference to the risk of incident disability. In other words, the mere act of social participation appeared to capture most of the health benefits.

However, other studies examining the effects of participation in civic organizations on all-cause mortality, [4], [5], cognitive function, [5], and oral health status, [17] have not always shown a reduction in risk with participation. This suggests that the relationship between incident functional disability and social participation may vary depending on the type of organization, a possibility that has yet to be studied.

To our knowledge, no studies have been conducted on the relationship between incident functional disability in older individuals and participation in organizations by type of organization or number of types of organizations. Clarifying whether or not participation in a diversity of groups reduces the risk of incident functional disability for example, can provide important hints for health promotion. For example, should health promotion agencies encourage participation in any and all types of organizations, or particular types? We therefore set out to examine the relationship between incident functional disability and social participation from the perspective of number of types of organizations and type of social participation in a prospective cohort study.

## Methods

### Study sample

The present study is based on the Aichi Gerontological Evaluation Study (AGES) Cohort Study data, [20], [21]. AGES is a part of Japan Gerontological Evaluation Study (JAGES). This study involves investigating factors associated with incident functional disability among non-institutionalized individuals aged 65 years and older. The region studied covers 6 municipalities in the Chita Peninsula of Aichi Prefecture, Japan (Handa city, Tokoname city, Agui town, Taketoyo town, Minamichita town and Mihama town). In October 2003, self-reported questionnaires were mailed to 29,374 community-dwelling individuals aged 65 years and older who were not eligible to receive benefits from public long-term care insurance (LTCI) services. The survey was conducted using a random sampling method stratified by region, age, and sex in the 2 larger municipalities (Handa city and Tokoname city) and a complete census (complete enumeration) of the 4 smaller municipalities (Agui town, Mihama town, Minami-Chita town, and Taketoyo town). For Handa and Tokoname city, we obtained a list of randomly selected eligible residents from the each municipal administration. Random sampling was stratified by three variables: by school district as a regional variable, by five-year age block (with 95 and older as the final block) and by sex. The official residential registries were maintained by the municipal administrations, and the Japanese registries included information such as age and sex. Questionnaires were sent to 5,000 people each in Handa city and Tokoname city and to all eligible people in the other municipalities (total questionnaires mailed = 28,152; men 45.8%). Response rates were as follows: Handa city 55.5%, Tokoname city 52.4%, Agui town 55.6%, Mihama town 49.8%, Minami-Chita town 51.1% and Taketoyo town 51.4%. Of those, 13,310 individuals (6,508 men; 6,802 women) agreed to participate, and thus formed the baseline of the AGES Cohort. They were followed for a 4-year period starting in November 2003 (observation period: November 2003 to October 2007). Analysis was carried out on 12,951 subjects, excluding 359 people whose information on age or sex was missing. The subjects that were excluded were those whose response on age in the questionnaire was 4 or more years off from the age recorded in the LTCI database maintained by the municipalities or whose response on sex in the questionnaire was different from that in the database, possibly because a family member or other person had completed the questionnaire on the subject's behalf. For analysis, the age and sex recorded in the LTCI database maintained by the municipalities were used. Subjects consisted of 6,320 men (48.8%) and 6,631 women (51.2%), with a mean age of 72.7±5.9 years.

### Incident functional disability

Incident functional disability was defined based on medical certification. Certification of disability is based on formal evaluation of the need for long-term care according to uniform criteria applied throughout Japan and based on both a home-visit interview as well as a written opinion from a primary physician, [22]. We obtained information on certification of needed long-term care, death, and moving out of the study area from the long-term care insurance database maintained by the municipalities.

### Social participation

For the purposes of this study, social participation was classified into eight types: neighborhood associations/senior citizen clubs/fire-fighting teams (local community), hobby groups (hobby), sports groups or clubs (sports), political organizations or groups (politics), industrial or trade associations (industry), religious organizations or groups (religion), volunteer groups (volunteer), and citizen or consumer groups (citizen). Subjects were given a choice between ‘currently participate’ or ‘do not participate’ for each type of organization. The total number of types of organizations in which each subject participated was tallied and participation was categorized as 0 (no participation), 1, 2 or ≥3 organizations or missing. No response for even one organization was deemed as missing. The 8 types of organizations were chosen on the basis of those used in the Japan General Social Survey (JGSS), [23], but the AGES Cohort Study added local community organizations as a type of local community organization, [20]. Organizations that are especially characteristic of Japan among the above types are senior citizen clubs and religious organizations. Japanese senior citizen clubs conduct wide-ranging activities, including group activities, such as sports, hobbies, cultural activities and performing arts, [20]. These clubs have conducted their activities in cooperation with local government welfare departments and/or similar public agencies. The majority of Japanese religious organizations are Shinto (39.3%) or Buddhist (38.6%). Other religious organizations include Christian and other faith communities (22.1%).

### Covariates

Based on previous studies, [19], age, sex, annual equivalized income, educational attainment, marital status, occupational status and self-reported medical conditions were used as covariates that may correlate with social participation and incident functional disability. Since behavioral, psychosocial, and physiological pathways may be potential mechanisms for social participation to influence health, [24], these factors were used to test which aspect of social participation accounts for the prevention of incident functional disability. Smoking, alcohol consumption, walking time, and frequency of going outdoors were used as behavioral factors. Depression (Geriatric Depression Scale: GDS), [25], emotional support, instrumental support and frequency of meeting friends were used as psychosocial factors. IADL, [26] was used as a physiological factor. All variables were set as dummy variables. A “missing” category was used in analysis to account for missing values in response to questions.

### Statistical analysis

Cox proportional hazards model was used to calculate the hazard ratio (HR) of incident functional disability over 4 years. Respondents who were lost to follow-up by moving or who died without incident functional disability were censored. In each model, non-participation in a organization was set as the referent category. Regression analysis was performed with simultaneous forced entry of age, sex, annual equivalized income, educational attainment, marital status, occupational status, and self-reported medical conditions as covariates (Model 1). In addition, participation in all 8 organizations was added only for “type of social participation.”

To test which aspect of social participation accounted for the prevention of incident functional disability, we added behavioral, psychosocial, or physiological factors sequentially to each model from Model 2 to Model 4 and inspected the change in the HR estimate associated with social participation. In Model 2, smoking, alcohol consumption, walking time, and frequency of going outdoors were added to the variables in Model 1. In Model 3, depression, emotional support, instrumental support, and frequency of meeting friends were added to the variables in Model 1. In Model 4, IADL was added to the variables in Model 1. We added all three classes of mediators in the final Model 5.

SPSS 21.0J was used for statistical analysis with a significance level of 5%.

Ethical approval for the study was obtained from the Nihon Fukushi University Ethics Committee.

## Results

Baseline characteristics are shown in Table 1. Younger ages and males tend to participate in more organizations. For type of social participation, over half of all respondents participated in a local community organization. The next commonest form of participation was in hobby groups, followed by sports organizations. The age of participants was lower than the mean for all respondents (72.7 years) for most types of organizations, except for local community and religion organizations. The ratio of men to women was roughly fifty-fifty for most types of organizations, but was over 70% for industry and politics organizations. The “missing” category was characterized by a higher mean age and smaller male-to-female ratio than the other categories.

**Table 1:** Baseline characteristics.

		N	%	Age	Men (%)
**Total**					
		12,951	100.0	72.7±5.9	48.8
**Equivalized income**					
Low		4,291	33.1	72.2±5.7	50.9
Middle		4,708	36.4	71.9±5.6	57.7
High		1,218	9.4	72.7±6.2	53.0
Missing		2,734	21.1	74.5±6.1	28.3
**Educational attainment**					
< 6 yrs		541	4.2	78.7±6.8	26.2
6–9 yrs		6,810	52.6	72.5±5.8	47.2
10–12 yrs		3,623	28.0	72.1±5.5	47.1
≥ 13 yrs		1,194	9.2	71.5±5.6	72.6
Missing		783	6.0	73.8±6.1	49.7
**Marital status**					
Married		8,659	66.9	71.5±5.1	60.8
Single		3,462	26.7	75.4±6.6	18.9
Missing		830	6.4	73.6±6.0	48.4
**Occupational status**					
Employed		3,198	24.7	70.3±4.8	63.6
Not employed		9,493	73.3	73.4±6.0	44.0
Missing		260	2.0	73.8±6.3	41.5
**Self-reported medical conditions**					
No illness or disability		2,155	16.6	71.0±5.6	52.3
Illness or disability that does not require treatment		1,249	9.6	72.2±5.9	57.6
Decided to discontinue treatment		826	6.4	72.5±5.9	47.3
In treatment		8,081	62.4	73.1±5.8	47.6
Missing		640	4.9	73.8±6.3	37.3
**Smoking**					
Never smoked		7,602	58.7	72.9±6.1	21.9
Past smoker		3,169	24.5	72.5±5.5	93.1
Current smoker		1,619	12.5	71.6±5.4	91.2
Missing		561	4.3	73.8±5.4	41.2
**Alcohol consumption**					
Non-drinker		8,340	64.4	73.3±6.1	31.8
Non-daily drinker		1,710	13.2	71.3±5.3	66.5
Daily drinker		2,640	20.4	71.3±5.1	92.1
Missing		261	2.0	75.1±6.5	39.1
**Walking time (per day)**					
< 30 min		4,191	32.4	73.1±6.0	49.1
30–60 min		4,222	32.6	72.5±5.8	51.5
60–90 min		1,616	12.5	72.5±5.7	54.5
> 90 min		1,529	11.8	71.8±5.9	50.1
Missing		1,393	10.8	73.0±6.2	31.6
**Frequency of going outdoors**					
Almost daily		5,760	44.5	71.6±5.4	49.5
2–3 times/week		4,033	31.1	73.0±5.8	46.1
About once/week		2,244	17.3	73.8±6.2	50.0
Rarely		417	3.2	75.9±7.5	57.1
Missing		497	3.8	74.1±6.4	50.1
**Depression**					
No depression		7,325	56.6	72.1±5.7	52.0
Depressive tendency		2,410	18.6	73.0±6.0	48.3
Depression		718	5.5	72.9±6.0	49.0
Missing		2,498	19.3	73.9±6.2	39.8
**Emotional support**					
Available		10,966	84.7	72.5±5.8	46.6
Not available		1,263	9.8	73.2±6.0	67.8
Missing		722	5.6	74.5±6.2	48.5
**Instrumental support**					
Available		11,630	89.8	72.6±5.9	49.9
Not available		726	5.6	72.4±5.5	34.2
Missing		595	4.6	73.7±5.8	45.7
**Frequency of meeting friends**					
Almost daily		2,434	18.8	72.6±5.6	39.2
2–3 times/week		3,198	24.7	72.3±5.6	40.9
About once/week		1,946	15.0	72.3±5.7	47.9
1–2 times/month		1,822	14.1	72.3±5.8	56.6
A few times a year or less		3,159	24.4	73.2±6.3	61.2
Missing		392	3.0	74.7±6.7	41.3
**IADL**					
High		5,378	41.5	71.5±5.0	45.3
Middle		2,633	20.3	72.0±5.5	53.9
Low		3,897	30.1	74.9±6.7	52.4
Missing		1,043	8.1	75.0±6.2	40.4
**Number of types of organizations**					
0		3,190	24.6	72.7±6.2	48.2
1		3,184	24.6	72.9±6.0	48.5
2		2,135	16.5	72.1±5.5	52.0
≥3		2,102	16.2	71.1±5.0	54.9
missing		2,340	18.1	74.1±6.2	41.7
**Type of social participation**					
Local community	participation	6,851	52.9	72.8±5.7	48.9
	non-participation	4,948	38.2	72.1±6.0	49.7
	missing	1,152	8.9	74.5±6.3	44.4
Hobby	participation	3,557	27.5	71.6±5.1	43.4
	non-participation	7,799	60.2	72.7±6.0	52.3
	missing	1,595	12.3	74.6±6.2	43.5
Sports	participation	2,373	18.3	70.8±4.9	52.6
	non-participation	8,860	68.4	72.8±6.0	48.9
	missing	1,718	13.3	74.5±6.1	43.0
Religion	participation	1,394	10.8	73.3±5.9	51.4
	non-participation	9,913	76.5	72.3±5.8	49.3
	missing	1,644	12.7	74.3±6.0	43.9
Industry	participation	1,199	9.3	71.6±5.4	77.4
	non-participation	10,124	78.2	72.5±5.9	46.8
	missing	1,628	12.6	74.5±6.0	40.4
Volunteer	participation	1,180	9.1	70.5±4.6	52.4
	non-participation	10,111	78.1	72.6±5.9	49.3
	missing	1,660	12.8	74.6±6.1	43.2
Politics	participation	921	7.1	71.8±5.4	70.5
	non-participation	10,573	81.6	72.5±5.9	48.1
	missing	1,457	11.3	74.6±6.0	40.3
Citizen	participation	532	4.1	71.0±4.7	43.0
	non-participation	10,659	82.3	72.5±5.9	49.9
	missing	1,760	13.6	74.4±6.1	43.6

Of the 12,951 respondents analyzed, 1,009 died, 1,528 became eligible for long-term care (i.e. became functionally disabled, according to our definition), and 140 moved out of the study area during the four year follow-up period. The incident rates of functional disability found by dividing the number of new cases by the number of follow-up years are shown in Table 2. The incident rate of functional disability decreased as the number of types of organizations increased, and was smallest for sports organizations, followed by volunteer, then hobby organizations.

**Table 2:** Incident rate of functional disability for 4 years.

		N	Incident/Person year	Incident rate
**Total**				
		12,951	1,528/47,453	0.032
**Number of types of organizations**				
0		3,190	447/11,391	0.039
1		3,184	390/11,624	0.034
2		2,135	192/8,010	0.024
≥3		2,102	124/8,021	0.015
missing		2,340	375/8408	0.045
**Type of social participation**				
Local community	participation	6,851	715/25,395	0.028
	non-participation	4,948	589/18,004	0.033
	missing	1,152	224/4,054	0.055
Hobby	participation	3,557	253/13,528	0.019
	non-participation	7,799	987/28,261	0.035
	missing	1,595	288/5,665	0.051
Sports	participation	2,373	128/9,139	0.014
	non-participation	8,860	1,103/32,191	0.034
	missing	1718	297/6,124	0.048
Religion	participation	1,394	180/5,098	0.035
	non-participation	9,913	1,069/36,469	0.029
	missing	1,644	279/5,886	0.047
Industry	participation	1,199	96/4,472	0.021
	non-participation	10,124	1,145/37,160	0.031
	missing	1,628	287/5,821	0.049
Volunteer	participation	1,180	74/4,486	0.016
	non-participation	10,111	1,161/37,055	0.031
	missing	1,660	293/5,913	0.050
Politics	participation	921	90/3,417	0.026
	non-participation	10,573	1,170/38,854	0.030
	missing	1,457	268/5,183	0.052
Citizen	participation	532	45/1,999	0.023
	non-participation	10,659	1,177/39,169	0.030
	missing	1,760	306/6,286	0.049

The results of Cox proportional-hazards model analysis of social participation and incident functional disability are shown in Table 3. In the crude model, participation was strongly protective of incident disability. Moreover, a “dose-response” relationship was seen, with progressively lower HRs as the number of different types of organizations increased. Regarding type of social participation, religion organizations was associated with an increased risk of disability without any covariate adjustment. In Model 1, where the data was adjusted for age, sex, annual equivalized income, educational attainment, marital status, occupational status, self-reported medical conditions, and participation in all 8 organizations (only for “type of social participation”), the hazard ratios became statistically significant for one or more different types of organizations HRs, and local community, hobby, and sports organizations HRs. Trend analysis of the data set omitting the missing categories for number of types of organizations yielded a p for liner trend of <0.01.

**Table 3:** Adjusted hazard ratios (95% confidence intervals) for incident functional disability.

	Crude		Model 1 a)		Model 2 b)		Model 3 c)		Model 4d)		Model 5e)	
	HR	95%CI	HR	95%CI	HR	95%CI	HR	95%CI	HR	95%CI	HR	95%CI
**Number of types of organizations**												
0	1.00		1.00		1.00		1.00		1.00		1.00	
1	0.85*	0.75–0.98	0.83*	0.73–0.95	0.87	0.76–1.00	0.89	0.78–1.02	0.88	0.77–1.01	0.93	0.81–1.07
2	0.61*	0.51–0.72	0.72*	0.61–0.85	0.79*	0.66–0.93	0.82*	0.69–0.98	0.82*	0.69–0.98	0.91	0.76–1.09
≥3	0.39*	0.32–0.48	0.57*	0.46–0.70	0.63*	0.52–0.78	0.69*	0.56–0.86	0.68*	0.55–0.83	0.78*	0.63–0.96
												
**Type of social participation** (reference: non-participation of each organization)												
Local community	0.86*	0.77–0.96	0.85*	0.76–0.96	0.89*	0.79–1.00	0.90	0.80–1.02	0.89*	0.79–1.00	0.94	0.83–1.06
Hobby	0.53*	0.46–0.61	0.75*	0.64–0.87	0.78*	0.66–0.91	0.80*	0.69–0.94	0.82*	0.70–0.96	0.85*	0.73–1.00
Sports	0.41*	0.34–0.49	0.66*	0.54–0.81	0.69*	0.56–0.85	0.71*	0.58–0.87	0.69*	0.56–0.84	0.73*	0.59–0.90
Religion	1.20*	1.03–1.41	1.07	0.90–1.27	1.08	0.91–1.28	1.06	0.89–1.26	1.10	0.93–1.31	1.09	0.92–1.30
Industry	0.70*	0.57–0.86	1.07	0.84–1.35	1.08	0.85–1.37	1.11	0.87–1.41	1.09	0.86–1.39	1.13	0.88–1.44
Volunteer	0.53*	0.42–0.66	0.92	0.70–1.20	0.94	0.72–1.23	0.95	0.73–1.25	0.98	0.74–1.28	0.99	0.76–1.30
Politics	0.87	0.71–1.08	1.18	0.93–1.50	1.19	0.94–1.52	1.21	0.95–1.54	1.24	0.98–1.58	1.24	0.97–1.58
Citizen	0.75	0.56–1.01	1.33	0.96–1.84	1.32	0.95–1.83	1.37	0.99–1.90	1.36	0.98–1.89	1.36	0.98–1.90

a) Model 1 is adjusted for age, sex, annual equivalized income, educational attainment, marital status, occupational status and self-reported medical conditions. In addition, participation in all 8 organizations is added only for “**type of social participation**”.

b) Model 2 is adjusted for the covariates in Model 1 plus smoking, alcohol consumption, walking time and frequency of going outdoors.

c) Model 3 is adjusted for the covariates in Model 1 plus depression, emotional support, instrumental support and frequency of meeting friends.

d) Model 4 is adjusted for the covariates in Model 1 plus IADL.

e) Model 5 is adjusted for the covariates in Model 1 plus smoking, alcohol consumption, walking time, frequency of going outdoors, depression, emotional support, instrumental support, frequency of meeting friends and IADL.

* Statistically significant variable (p<0.05).

In sub-analysis, we sought to check for the possibility of reverse causation (*i.e.* the possibility that people who were feeling unwell selectively participated). We did this by excluding the 366 subjects who became certified for long-term care within the first year of follow-up. The HRs in this sensitivity analysis were 0.86 (0.73–1.00) for participation in one, 0.74 (0.60–0.89) for participation in two, and 0.53 (0.42–0.68) for participation in three or more different types of organizations, with the significant difference disappearing only for participation in one type of organization. In addition, the HR for participation in a local community organization was 0.84 (0.73–0.96), in a hobby organization was 0.76 (0.63–0.90) and in a sports organization was 0.72 (0.57–0.90).

We also stratified the HRs by gender. For men, the HRs were 0.84 (0.67–1.05) for participation in one, 0.80 (0.62–1.04) for participation in two, and 0.66 (0.50–0.87) for participation in three or more different types of organizations. The HR for participation in a local community organization was 0.87 (0.72–1.05), in a hobby organization was 0.74 (0.58–0.94) and in a sports organization was 0.64 (0.48–0.85). For women, the HRs were 0.85 (0.71–1.01) for participation in one, 0.67 (0.52–0.83) for participation in two, and 0.50 (0.37–0.68) for participation in three or more different types of organizations. The HR for participation in a local community organization was 0.86 (0.74–1.01), in a hobby organization was 0.73 (0.60–0.90) and in a sports organization was 0.66 (0.49–0.88). No significant differences were seen in either men or women in any other types of participation in organizations.

Next, to test which aspect of social participation accounts for the prevention of incident functional disability, we added behavioral, psychosocial, or physiological factors to each model from Model 2 to Model 4. For each model, the HRs for types of participation that were significantly different in Model 1 tended towards 1.00. In Model 5 adjusted for these behavioral, psychosocial, or physiological factors, there were no differences between non-participation and participation in one or two types of organizations or in a local community organization.

## Discussion

In this study, we examined the relationship between incident functional disability and social participation from the perspective of number of types of organizations and type of social participation in a prospective cohort study. Social participation significantly lowered the risk of incident functional disability, and this effect increased with an increasing variety of organizations in which subjects participated. In analyses stratified by gender, as well as excluding subjects who became disabled within one year of follow-up, the results still suggested a protective effect of participating in more than two organizations. Previous studies have suggested that social participation can lower the risk of incident disability, [10], [11], which supports the results of the present study. Analysis adjusted for behavioral, psychosocial, and physiological factors resulted in a tendency towards weaker positive associations. Behavioral, psychosocial, and physiological pathways may be potential mechanisms for social participation to influence health, [24]. The results of the present study support that finding. In addition, participation in a greater number of different organizations brought the HR closer to 1.00. Participation in multiple organizations may have a protective effect on depression, [15], well-being, [16] and oral health status, [17], while having a role within the organization may reduce the risk of mental health problems, [18]. These findings indicate that social participation may decrease the risk of incident functional disability in older Japanese people, and that this effect may be strengthened by participation in a variety of organizations.

The relationship between social participation and prevention of functional decline may be under-pinned by at least three distinct mechanisms. First, social participation encourages older individuals to keep active (e.g. getting dressed each day to leave the house), and these daily routines may help to preserve functioning (the “use it or lose it” hypothesis). Secondly, social participation provides individuals with access to various forms of social support (e.g. access to material resources, or health-relevant information) which may promote the preservation of functional status. Thirdly, social participation may have direct physiological benefits such as buffering stress, boosting host resistance, and lowering biomarkers of disease risk, such as inflammation, [27].

Regarding type of social participation, the risk was significantly lower for participation in a local community, hobby, or sports organization. This decrease remained significant even when those who became certified for long-term care within a year were excluded from the analysis. When each gender was examined separately, the HR remained lower than 1.00 for participation in a local community organization, but this difference was no longer significant. Participation in a hobby or sport organization significantly lowered the risk for both men and women. In a similar trend, previous studies that each looked at different organizations did not always show participation to lower the risk of all-cause mortality, [4], [5] or impaired cognitive function, [5]. The finding that participation in a hobby or sports organization significantly lowers the risk for both men and women is similar to the finding of an association with all-cause mortality, [4]. Among the different types of social participation, it is thus likely that participating in a local community, hobby, or sports group organization may be especially effective for decreasing the risk of requiring long-term care in the future.

For these three types of organizations, adjusting for behavioral, psychosocial, and physiological factors resulted in a tendency towards a weaker positive relationship. The association with participation in a hobby or sports organization may be due to the influence of hobby activities, [28] or physical activities, [29] that may help prevent incident functional disability. In addition to the benefits of physical activity, participation in a sports organization may have positive effects from the social interaction itself, e.g. instrumental and emotional support exchanged between members, or even just the social reinforcement (i.e. reinforcers such as acceptance, praise, and attention from other people) that comes from belonging to a group, [19]. The reason for the protective effect of local community organizations may be that sports and leisure activities are often included in the activities of these organizations, [20]. One type of neighborhood-based organization common throughout Japan is the community-based senior centers (called “salons”), which have been shown to improve self-rated health, [30] and increase emotional social support, [31]. These may help reduce the risk of needing long-term care. However, the association was weaker than that of participation in hobby and sports organizations, and was no longer significant when each gender was analyzed separately. The reason for this may be in the negative side of social relationships, such as sense of obligation to the community or burden, [32]. These findings point to the existence of behavioral, psychosocial, and physiological pathways in the weakening of the risk of incident functional disability due to participation in a local community, hobby, and sports organization. Significant associations remained for participation in three or more organizations and in participation in a hobby and sports organization even in Model 5 that included all covariates. Further studies are needed to elucidate the reason for this, as it is possible that there may be some other factor acting on these types of participation that were not examined here (such as eating behavior or physical fitness).

Of all types of participation, only participation in a religious group showed an inverse association with incident functional disability in the crude model, although the association became statistically non-significant in later models that adjusted for age and other variables. This result is contrary to a meta-analysis that found religious involvement to be related to low mortality, [33]. However, most of the studies in this topic have been conducted in western settings. Religious involvement has a very different connotation in Japanese society, where most people (84%) do not claim to adhere to any personal religion, [34]. We speculate that religious involvement in this context is more likely to be triggered by health problems, and hence the possibility of reverse causation is heightened.”

The present study has five limitations. First and foremost is that we cannot completely exclude the possibility of reverse causation, even though the study was longitudinal and none of the subjects had disability at the start of follow up. Although we carefully controlled for a number of covariates, and even conducted a sensitivity analysis excluding people who developed disability within one year of baseline, there is nonetheless a possibility of residual confounding by underlying health status (e.g. vitality, “pep”, or some other subtle difference in health that our measurements were unable to capture). Thus, subtle differences in the vitality of individuals (for example in lung capacity or cardiovascular fitness) could cause them to selectively participate in more organizations, as well as to select more vigorous activities (such as sports) over more sedentary activities (like chatting to neighbors). Causal inference in this situation is quite challenging. Given the expense involved in conducting randomized experiments, some have advocated the use of “natural” experiments, such as instrumental variables. In a recent study, Ichida et al., [29] used “distance to the nearest newly opened community center” as an instrument for social participation, The authors found that old people who happened to live closer to a newly opened community “salon” were more likely to participate, and that salon participation was associated with improved health over follow-up. A second limitation is that the term “local community organization” covered neighborhood associations, senior citizen clubs and fire-fighting teams. We were therefore unable to distinguish how participation in each of these three types of organizations related to incident functional disability. A third limitation is that we did not inquire about the frequency of participation in each organization. The frequency of meetings may vary greatly according to the type of organization, but the effects of variation in frequency are not reflected in this study. A fourth limitation is that we did not inquire about the level of involvement or commitment of the individual to the organization. The involvement of individuals in organizations may vary in intensity, e.g. according to whether they assume leadership roles or they choose to participate as regular members, [18]. Further studies are needed to examine these additional questions. A fifth and final limitation is that our response rate to the baseline invitation was only 46%, so that our findings may not be representative of the areas in Japan in which the surveys were conducted. However, a response rate of 40% is quite high by western standards (where the response to postal surveys can be as low as 10%). Moreover, depending on the objectives of the study, we need not insist on representativeness as an obligatory feature of a cohort study, [35].

In conclusion, social participation may lower the risk of incident functional disability in older Japanese people, and this effect may be strengthened by participation in a variety of organizations. Moreover, participating in a local community, hobby, or sports group or organization may be especially effective for decreasing the risk. These findings suggest that support for older Japanese people that encourage participation in a variety of organizations, centered on local community, hobby, and sports organizations, may be effective for preventing the need for long-term care.

## References

[1] Stuck AE, Walthert JM, Nikolaus T, Büla CJ, Hohmann C, et al (1999) Risk factors for functional status decline in community-living elderly people. Social Science & Medicine 48: 445–469. 10075171 10.1016/s0277-9536(98)00370-0

[2] Julianne HL, Timothy BS, Layton JBL (2010) Social relationships and mortality risk: a meta-analytic review. Plos Medicine 7(7): e1000316. 20668659 10.1371/journal.pmed.1000316 PMC2910600

[3] WHO. Active ageing: a policy framework. Available: http://whqlibdoc.who.int/hq/2002/WHO_NMH_NPH_02.8.pdf. Accessed 2013 Feb 28.

[4] Aida J, Kondo K, Hirai H, Subramanian SV, Murata C, et al (2011) Assesing the association between all-cause mortality and multiple aspects of individual social capital among the older Japanese. BMC Public Health 11: 499. 21702996 10.1186/1471-2458-11-499 PMC3144463

[5] Hsu HC (2007) Does social participation by the elderly reduce mortality and cognitive impairment? Aging & Mental Health 11: 699–707. 18074257 10.1080/13607860701366335

[6] Glass TA, Medes de Leon C, Marottoli RA, Berkman LF (1999) Population based study of social and productive activities as predictors of survival among elderly Americans. BMJ 319: 478–83. 10454399 10.1136/bmj.319.7208.478 PMC28199

[7] Väänänen A, Murray M, Koshinen A, Vahtera J, Kouvonen A, et al (2009) Engagement in cultural activities and cause-specific mortality: Prospective cohort study. Preventive Medicine 49: 142–147. 19589351 10.1016/j.ypmed.2009.06.026

[8] Iwasaki M, Otani T, Sunaga R, Miyazaki H, Liu Xiao, et al (2002) Social networks and mortality based on the Komo-Ise cohort study in Japan. International Journal of Epidemiology 31: 1208–1218. 12540724 10.1093/ije/31.6.1208

[9] Ali SM, Merlo J, Rosvall M, Lithman T, Lindström M (2006) Social capital, the miniaturization of community, traditionalism and first time acute myocardial infarction: A prospective cohort study in southern Sweden. Social Science & Medicine 63: 2204–2217. 16797808 10.1016/j.socscimed.2006.04.007

[10] James BD, Boyle PA, Buchman AS, Bennett DA (2011) Relation of late-life social activity with incident disability among community-dwelling older adults. The journals of gerontology. Series A, Biological Sciences and Medical Sciences 66(4): 467–473. 10.1093/gerona/glq231 PMC3055280 21300745

[11] Hirai H, Kondo K, Ojima T, Murata C (2009) Examination of risk factors for onset of certification of long-term care insurance in community-dwelling older people: AGES project 3-year follow-up study. Japanese Journal of Public Health 56(8): 501–512 (in Japanese) 19827611

[12] Buchman AS, Boyle PA, Wilson RS, Fleischman DA, Leurgans S, et al (2009) Association between late-life social activity and motor decline in older adults. Archives of Internal Medicine 169: 1139–1146. 19546415 10.1001/archinternmed.2009.135 PMC2775502

[13] James BD, Wilson RS, Barnes LL, Bennett DA (2011) Late-life social activity and cognitive decline in old age. Journal of International Neuropsychological Society 17: 998–1005. 10.1017/S1355617711000531 PMC3206295 22040898

[14] Ciao C, Weng LJ, Botticello AL (2011) Social participation reduces depressive symptoms among older adults: an 18-year longitudinal analysis in Taiwan. BMC Public Health 11: 292. 21569285 10.1186/1471-2458-11-292 PMC3103460

[15] Cruwys T, Dingle GA, Haslam C, Haslam SA, Jetten J, et al (2013) Social group memberships protect against future depression, alleviate depression symptoms and prevent depression relapse. Social Science & Medicine 98: 179–186. 24331897 10.1016/j.socscimed.2013.09.013

[16] Haslam C, Holme A, Haslam SA, Iyer A, Jetten J, et al (2008) Maintaining group memberships: Social identity continuity predicts well-being after stroke. Neuropsychological Rehabilitation 18(5/6): 671–691. 18924001 10.1080/09602010701643449

[17] Takeuchi K, Aida J, Kondo K, Osaka K (2013) Social participation and dental health status among older Japanese adults: a population-based cross-sectional study. Plos One 8(4): e61741. 23613921 10.1371/journal.pone.0061741 PMC3629217

[18] Takagi D, Kondo K, Kawachi I (2013) Social participation and mental health: moderating effects of gender, social role and rurality. BMC Public Health 13: 701. 23902596 10.1186/1471-2458-13-701 PMC3734140

[19] Kanamori S, Kai Y, Kondo K, Hirai H, Ichida Y, et al (2012) Participation in sports organizations and the prevention of functional disability in older Japanese: the AGES Cohort Study. Plos One 7(11): e51061. 23226458 10.1371/journal.pone.0051061 PMC3511372

[20] Kondo K (2010) Health Inequalities in Japan: An Empirical Study of Older People. Melbourne: Trans Pacific Press. 291P.

[21] Nishi A, Kondo K, Hirai H, Kawachi I (2011) Cohort Profile: The AGES 2003 cohort study in Aichi, Japan. Journal of Epidemiology 21(2): 151–157. 21325730 10.2188/jea.JE20100135 PMC3899507

[22] Tsutsui T, Muramatsu N (2005) Care-needs certification in the long-term care insurance system of Japan. Journal of the American Geriatrics Society 53(3): 522–527. 15743300 10.1111/j.1532-5415.2005.53175.x

[23] Osaka university commerce JGSS Research Center. JGSS no chousa gaiyou. Available: http://jgss.daishodai.ac.jp/surveys/sur_question.html. Accessed 2013 Aug 4.) (In Japanese).

[24] Umberson D, Montez JK (2010) Social relationships and health: a flashpoint for health policy. Journal of Health and Social Behavior 51(suppl): S54–S66. 20943583 10.1177/0022146510383501 PMC3150158

[25] Sheikh JI, Yesavage JA (1986) Clinical gerontology: a guide to assessment and intervention. London: Routledge. 532P.

[26] Koyano W, Shibata H, Nakazato K (1987) Prevalence of disability in instrumental activities of living among elderly Japanese. Japanese Journal of Public Health 34(3): 109–114 (In Japanese) 10.1093/geronj/43.2.s41 2964469

[27] Glei DA, Goldman N, Ryff CD, Lin YH, Weinstein M (2012) Social relationships and inflammatory markers: an analysis of Taiwan and the U.S.. Social Science & Medicine 74(12): 1891–1899. 22483707 10.1016/j.socscimed.2012.02.020 PMC3348237

[28] Takeda T, Kondo K, Hirai H (2010) Psychosocial risk factors involved in progressive dementia-associated senility among the elderly residing at home AGES-Three year cohort longitudinal study. Japanese Journal of Public Health 57(12): 1054–1065 (In Japanese) 21348280

[29] Paterson DH, Warburton DE (2010) Physical activity and functional limitations in older adults: a systematic review related to Canada's Physical Activity Guidelines. The International Journal of Behavioral Nutrition and Physical Activity 7: 38–59. 20459782 10.1186/1479-5868-7-38 PMC2882898

[30] Ichida Y, Hirai H, Kondo K, Kawachi I, Takeda T, et al (2013) Does social participation improve self-rated health in the older population? A quasi-experimental intervention study. Social Science & Medicine 94: 83–90. 23931949 10.1016/j.socscimed.2013.05.006

[31] Takeda T, Kondo K, Hirai H (2009) Preventive intervention of senile dementia focusing on psychosocial factors: Intervention theory based on the population health approach and its evaluation of midterm outcomes (In Japanese). The Journal of Japanese Occupational Therapy Association 28 (2): 178–186.

[32] Rook KS (1984) The negative side of social interaction: impact on psychological well-being. Journal of Personality and Social Psychology 46: 1097–1108. 6737206 10.1037//0022-3514.46.5.1097

[33] McCullough ME, Hoyt WT, Larson DB, Koenig HG, Thoresen C (2000) Religious involvement and mortality: a meta-analytic review. Health Psychology 19(3): 211. 10868765 10.1037//0278-6133.19.3.211

[34] Earhart, H Byron (1974) Japanese Religion: Unity and Diversity. Encino, Calif: Dickenson Publishing Co. 148 p.

[35] Rothman KJ, Gallacher JE, Hatch EE (2013) Why representativeness should be avoided. International Journal of Epidemiology 42: 1012–1014. 24062287 10.1093/ije/dys223 PMC3888189

